# Postbiotic Intervention in Sarcopenia: The Role of *Lactiplantibacillus plantarum* HY7715 and Its Extracellular Vesicles

**DOI:** 10.3390/life15071101

**Published:** 2025-07-14

**Authors:** Kippeum Lee, Soo Dong Park, Joo Yun Kim, Jae Jung Shim, Jae Hwan Lee

**Affiliations:** R&BD Center, Hy Co., Ltd., 22 Giheungdanji-ro 24 Beon-gil, Giheung-gu, Yongin-si 17086, Republic of Korea; joy4917@hanmail.net (K.L.); soodpark@hy.co.kr (S.D.P.); jjshim@hy.co.kr (J.J.S.); jaehwan@hy.co.kr (J.H.L.)

**Keywords:** postbiotics, *Lactiplantibacillus plantarum* HY7715, extracellular vesicle, sarcopenia, gut barrier impairment

## Abstract

Sarcopenia, the age-related loss of skeletal muscle mass and function, is associated with inflammation, mitochondrial dysfunction, and gut barrier impairment. This study investigates the postbiotic effects of heat-killed *Lactiplantibacillus plantarum* HY7715 (HY7715) and its extracellular vesicles (EVs) on muscle health and intestinal integrity. In C2C12 myotubes, both treatments enhanced myogenic differentiation by upregulating Myf5 and MYOG, and improved mitochondrial activity and biogenesis via increased PGC1α and mTOR expression. Under TNFα-induced muscle atrophy, they suppressed expression of atrophy-related markers (Fbox32, MuRF1, and myostatin). EVs showed stronger anti-inflammatory effects by reducing IL6 expression in muscle cells. In intestinal Caco-2 cells, HY7715-derived EVs improved barrier function by upregulating tight junction proteins (ZO-1, occludin, and claudins), and effectively reduced LPS-induced inflammation. These findings suggest that heat-killed HY7715 and its EVs may alleviate sarcopenia by enhancing muscle regeneration and maintaining intestinal homeostasis, highlighting their potential as safe, gut–muscle axis-targeting postbiotic interventions for healthy aging.

## 1. Introduction

Myogenesis is the process through which myofibrils undergo morphological and functional changes in response to internal or external stimuli [1]. Skeletal muscle is primarily composed of myofibrils—large, cylindrical, multinucleated cells—formed via sequential processes including cell differentiation, fusion, migration, and cross-linking [2]. Accounting for approximately 40% of the total body mass, skeletal muscle plays essential roles in voluntary movement and metabolic homeostasis [3].

With aging, progressive loss of skeletal muscle mass and function (referred to as sarcopenia) commonly occurs and is a major contributor to frailty and physical decline [4,5]. While the pathogenesis of sarcopenia is multifactorial, mitochondrial dysfunction and degradation of muscle protein quality are considered principal factors [6,7]. In particular, increased levels of protein degradation-related markers such as muscle RING Finger 1 (MuRF-1) and F-box only protein 32 (Fbxo32, also known as Muscle Atrophy F-box) contribute to the occurrence of sarcopenia by elevating degradation, leading to muscle atrophy [7]. In addition, impaired mitochondrial quality control has been linked to reduced muscle strength and regenerative capacity [8]. Mitochondria are critical for ATP synthesis and contribute to muscle endurance and recovery [9,10]. Numerous studies have shown that mitochondrial dysfunction is involved in the progression of sarcopenia through decreased muscle function [11,12].

Recent studies have shown that certain probiotic strains can alleviate sarcopenic symptoms [13,14]. Although physical exercise remains the most effective intervention, dietary supplementation offers a feasible alternative for individuals with limited mobility [15,16]. Recently, natural probiotics have been reported to reduce muscle loss and improve strength in older adults [17,18,19]. *Lactiplantibacillus plantarum* HY7715 is a probiotic strain that exhibits high bile acid resistance and notable riboflavin (vitamin B2) production, according to our previous studies [20]. Our previous work demonstrated that *L. plantarum* HY7715 (HY7715) prevented muscle atrophy and improved strength in 80-week-old aged Balb/c male mice. In addition, HY7715 recovered the microbiome composition and beta-diversity shift [21]. However, the bioactive components responsible for these effects remain unidentified.

Inactivated probiotics (parabiotics) and their metabolites (postbiotics) are gaining attention due to their safety and physiological activity [22,23]. Recent studies have also reported that heat-killed lactic acid bacterial strains have health-promoting effects [24,25]. However, no study has investigated whether heat-treated HY7715 affects myogenesis and mitochondrial biogenesis in muscle cells. On the other hand, probiotic-derived extracellular vesicles (EVs) may play an important role as functional substances. EVs are lipid-bilayer-bound nanoparticles (30–150 nm) that mediate intercellular and interspecies communication [26,27]. Gut microbiota-derived EVs contribute to host tissue regulation, including muscle and immune function. Despite this, the effects of HY7715-derived EVs on myogenesis and muscle atrophy remain unexplored. Therefore, this study aims to investigate whether EVs isolated from HY7715 can mitigate TNFα-induced atrophy in muscle cells and influence gut–muscle axis signaling using an intestinal epithelial model. Therefore, we investigated whether heat-treated HY7715 could be used as a potential therapeutic agent for sarcopenia in cell experiments. In addition, this study aims to investigate whether EVs isolated from HY7715 could attenuate TNFα-induced muscle cell atrophy and affect gut–muscle axis signaling using an intestinal epithelial model.

## 2. Materials and Methods

### 2.1. Preparation of Heat-Killed Probiotic

HY7715 was isolated from kimchi and stored in a seed culture library at hy Co., Ltd. (Yongin-si, Republic of Korea). It was cultured in de Man, Rogosa, and Sharp broth medium (MRS, Difco Corp., Sparks, MD, USA) in a fermenter for 15–18 h at 37 °C; then, the culture was centrifuged at 8000× *g* and 4 °C for 15 min. HY7715 was then serially diluted to calculate the number of colony-forming units (CFUs) obtained for subsequent in vitro studies. HY7715 was then centrifuged at 2000× *g* for 10 min and washed twice with phosphate-buffered saline (PBS); the pellets were resuspended in PBS pH 7.2 to prepare a 10^9^ CFU/mL stock solution. The bacterial stocks were killed by exposure to a temperature of 100 °C for 30 min and then harvested by centrifugation. Finally, the whole-cell lysates were used for further experiments, in which the *L. plantarum* type strain KCTC3108 (KCTC3108) was used as a control. KCTC3108 probiotic was prepared in the same way as HY7715.

### 2.2. Preparation of HY7715-Derived EVs

EVs were isolated from the culture supernatant of heat-killed HY7715. Briefly, the culture supernatant was obtained by sequential centrifugation. HY7715 culture medium was first centrifuged at 2000× *g* for 15 min at 4 °C to remove cell debris, and then the supernatants were transferred to new tubes and mixed with a commercial total exosome isolation reagent (Cat. no. 4478359; Thermo Fisher Scientific, Waltham, MA, USA) overnight. HY7715 EV pellets were prepared by high-speed centrifugation at 10,000× *g* for 60 min at 4 °C). The HY7715-derived EV samples were then resuspended in PBS to a final stock concentration of 1 mg/mL and stored at −80 °C. KCTC3108 EVs, which were used as a control, were prepared in the same way. The quantities of EVs obtained were estimated by measuring the total protein concentrations using a DC protein assay kit (Bio-Rad, Hercules, CA, USA), in accordance with the manufacturer’s instructions. The absorbances were measured at 750 nm using a microplate reader (BioTek, Winooski, VT, USA).

### 2.3. Analysis of the EVs

The size distributions of the HY7715-derived EVs were assessed using a Nanosight NS300 instrument (Malvern Panalytical Ltd., Malvern, UK; version no. 3.4). NanoSight NTA 3.4 analytical software was used to accurately quantify the sizes and concentrations of the HY7715-derived EVs in each sample in triplicate.

### 2.4. Cell Culture and EVs

Skeletal muscle C2C12 myoblasts (CRL-1772) were obtained from the American Type Culture Collection (Manassas, VA, USA) and maintained in DMEM medium supplemented with 10% fetal bovine serum (Gibco, Thermo Fisher Scientific, Waltham, MA, USA), 1% antibiotic–antimycotic solution (Thermo Fisher Scientific), and 3.7 g/L sodium bicarbonate in a 5% CO_2_-containing humidified incubator at 37 °C. When the myoblast reached 80% confluence, the medium was replaced with DMEM containing 2% equine serum (Gibco, Thermo Fisher Scientific), and differentiation occurred over 5 days. To induce atrophy of the C2C12 cells, 100 ng/mL TNFα was added during the period of differentiation. Ursolic acid (UA, 1 mM), which has been reported to ameliorate sarcopenia, was used as a positive control. Heat-killed HY7715 probiotic (10^6^ CFU/mL) and EVs isolated from HY7715 were diluted to final concentrations of 1 μg/mL, and the control heat-killed KCTC3108 and the derived EVs were used at the same concentrations.

Caco-2 colorectal adenocarcinoma cells were obtained from the Korean Cell Line Bank (Seoul, Republic of Korea) and cultured in DMEM containing 1% P/S and 10% FBS at 37 °C in humidified air containing 5% CO_2_ for 18 days, to permit complete differentiation, with the growth medium being replaced every 3 days. To induce the dysfunction of tight junctions, 1 ng/mL lipopolysaccharide (LPS) was added to the medium for the final 4 h of the incubation.

### 2.5. Western Blot Analysis

The expression levels of proteins of interest in the C2C12 and Caco-2 cells were determined by Western blot analysis. The cells were harvested and homogenized at 4 °C in lysis buffer (iNtRON Biotechnology, Seoul, Republic of Korea), and then the total protein concentrations of the lysates were quantified using a protein assay kit (Bio-Rad). The lysates, each containing 15 μg total protein, were subjected to SDS–PAGE and then electro transferred to PVDF membranes. The membranes were blocked using 5% skim milk in Tris-buffered saline containing 0.1% Tween 20 (TBS-T) for 60 min, washed with TBS-T, and then incubated with primary antibodies overnight at 4 °C. They were then exposed to horseradish peroxidase-conjugated secondary antibodies, and the target protein bands were visualized using Pierce ™ ECL solution (Thermo Scientific, MA, USA). Antibodies targeting mouse myogenic factor 5 (Myf5), mechanistic target of rapamycin (mTOR), peroxisome proliferator-activated receptor coactivator 1 alpha (PGC1α), myostatin, and glyceraldehyde 3-phosphate dehydrogenase (GAPDH), and antibodies targeting human zonula occludens-1 (ZO-1), occludin (OCDN), claudin-1 (CLDN-1), and GAPDH were obtained from Cell Signaling Technology (Danvers, MA, USA).

### 2.6. RNA Isolation, cDNA Synthesis, and Gene Expression Analysis

RNA was extracted from the cells using easy-spin RNA Extraction Kit (iNtRON Biotechnology, Gyeonggi-do, Republic of Korea). cDNA was synthesized using Maxime RT PreMix (LiliF^TM^ Diagnostics, Seoul, Republic of Korea), involving incubations at 45 °C for 60 min and 95 °C for 5 min. The concentrations of the cDNA samples obtained were measured using NanoDrop 2000 (Thermo Scientific). Gene expression analysis was performed using QuantStudio 6-Flex Real-time PCR System (Applied Biosystems, Foster City, CA, USA), Gene Expression Master Mix (Applied Biosystems), and specific TaqMan probes (Applied Biosystems). The mouse TaqMan probes used were as follows: *Gapdh*, Mm99999915_g1; *Fbox32*, Mm00499523_m1; *Trim63*, Mm01185221_m1; *Myh1*, Mm01332489_m1; *Myog*, Mm00446194_m1; *Mtor*, Mm00444968_m1; and *Pgc1a*, Mm01208835_m1. The human TaqMan probes used were as follows: *GAPDH*, Hs03929097_g1; *ZO1*, Hs01551861_m1; *OCDN*, Hs00170162_m1; *CLDN4*, Hs00976831_s1; and *IL1B*, Hs01555410_m1. The expression of each target gene was calculated using the 2^−∆∆CT^ method and normalized to that of the reference gene *GAPDH*.

### 2.7. Mitochondrial Staining

Fully differentiated C2C12 myotubes were treated with TNFα in the presence or absence of heat-killed HY7715 or HY7715-derived EVs. Subsequently, the myotubes were incubated with 500 nM MitoTracker Deep Red for 30 min at 37 °C, washed with PBS, and fixed in 4% paraformaldehyde. The cells were then rinsed three times with PBS and photographed using a ZOE Fluorescent Cell Imager (Bio-Rad). The mitochondria and nuclei were stained using MitoTracker Deep Red FM (CS8778, Cell Signaling) and DAPI (D21490, Invitrogen, Waltham, MA, USA), respectively, and the intensity and area of MitoTracker staining was assessed using a fluorescence microscope and ImageJ software (version 1.46; National Institutes of Health, Bethesda, MD, USA).

### 2.8. Statistical Analysis

Statistical analysis was performed using SPSS (version 20.0, IBM, Inc., Armonk, NY, USA). The data are summarized using the mean ± standard deviation (SD). Datasets were compared using one-way ANOVA, followed by Duncan’s test. *p* < 0.05 was considered to indicate statistical significance.

## 3. Results

### 3.1. Analysis of Lactiplantibacillus plantarum HY7715-Derived EVs

**Figure 1:**
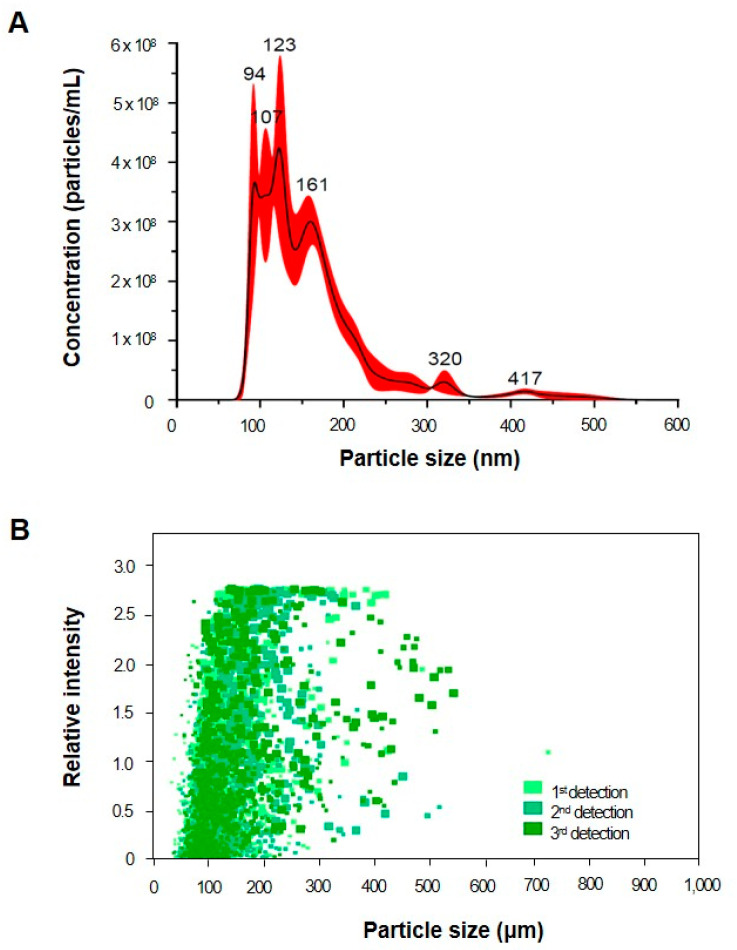
Characterization of HY7715-derived EVs including the particle concentration and diameters. (**A**) Nanoparticle tracking analysis of HY7715-derived EVs (*n* = 3). (**B**) Nanoparticle scattering intensity analysis of HY7715-derived EVs. The size distribution was evaluated using Malvern NanoSight NS300 and NanoSight NTA 3.4 software.

In our study, HY7715-derived EVs were characterized using nanoparticle tracking analysis, which revealed a particle concentration of 4.11 × 10^10^ particles/mL and modal and mean diameters of 127.6 ± 13.4 nm and 143.8 ± 1.1 nm, respectively (Figure 1). These data indicate that HY7715-derived EVs fall within the range of 10 nm to 300 nm in diameter, known as the bacterial membrane vesicle size range. To determine whether HY7715-derived EVs are one of the bioactive components of HY7715 parabiotics for improving sarcopenia, a concentration of 1 ng/mL was used in subsequent cell experiments.

### 3.2. Heat-Killed HY7715 Enhances Myoblast Differentiation and Suppresses Atrophy in C2C12 Myotubes

**Figure 2:**
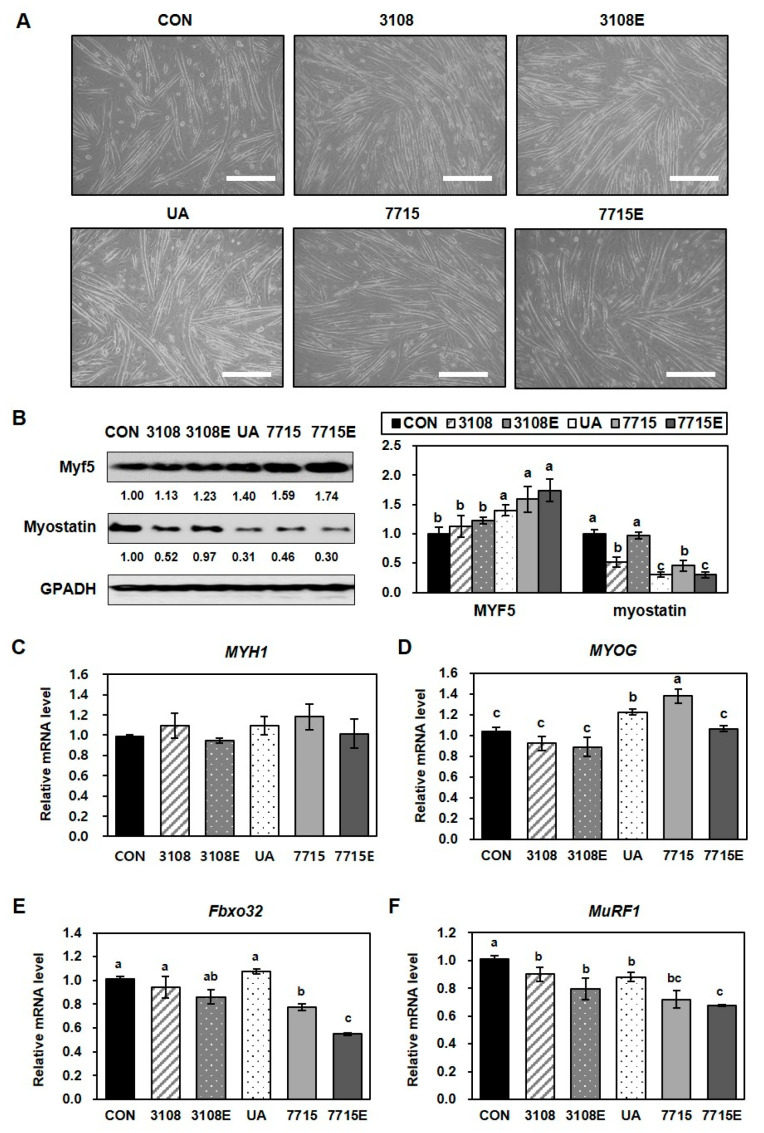
Effect of heat-killed *L. plantarum* HY7715 and its EVs on C2C12 myotube differentiation and atrophy. (**A**) C2C12 cells were differentiated for five days in the presence of 10^6^ CFU/mL of heat-killed HY7715 (7715), heat-killed KCTC3108 (3108), EVs derived from HY7715 (7715E), or KCTC3108 (3108E). Ursolic acid (1 mM, UA) served as a positive control. Bar: 100 μm. (**B**) Protein expression levels of Myf5 and myostatin assessed via Western blot. Expression was normalized to GAPDH, and the fold change relative to control is indicated. (**C**–**F**) Relative mRNA expression levels of MYH1, MYOG, Fbox32, and MuRF1 determined by qPCR. Data are expressed as mean ± SD (*n* = 4). Statistical analysis was performed using one-way ANOVA followed by Tukey’s multiple range test. Different letters indicate significant differences (*p* < 0.05).

Skeletal muscle atrophy is often linked to reduced expression of myogenic regulatory factors like Myf5 and myogenin, as well as enhanced protein degradation via the myostatin–ubiquitin–proteasome pathway [28,29]. To evaluate the potential of heat-killed HY7715 and its EVs in muscle regeneration, we assessed their effects—alongside those of heat-killed KCTC3108 (a reference strain), its EVs, and ursolic acid (UA, a positive control)—on C2C12 myoblast differentiation over five days. As shown in Figure 2A, C2C12 cells treated with heat-killed HY7715 and heat-killed KCTC3108 exhibited more advanced myotube morphology compared to controls. EVs from both strains also promoted differentiation.

To confirm the effects of heat-killed HY7715 and HY7715-derived EVs on myoblast differentiation and fusion, we analyzed the expression of signaling intermediates involved in myogenesis using Western blot analysis and qPCR. As shown in Figure 2B, the increase in Myf5 protein expression in myotubes treated with heat-killed HY7715 or HY7715-derived EVs was significantly larger (159% and 174%, respectively) than in control cells, but the increases in expression caused by treatment with KCTC3108- or KCTC3108-derived EVs was not significant (113% and 123%, respectively). Meanwhile, in cells treated with UA, heat-killed HY7715, or HY7715-derived EVs, the expression of myostatin was significantly lower than that in control cells (31%, 46%, and 30%, respectively). In cells treated with heat-killed KCTC3108 or KCTC3108-derived EVs, the protein expression of myostatin was lower (52% and 97%, respectively) than that of the control group, and the effect was smaller than that of HY7715 and other postbiotics. Western blot analysis revealed that Myf5 protein levels significantly increased in heat-killed HY7715 (159%) and HY7715-EV-treated cells (174%), whereas heat-killed KCTC3108 and its EVs had weaker effects (113% and 123%, respectively). Conversely, myostatin levels were markedly reduced in UA (0.31-fold), heat-killed HY7715 (0.46-fold), and HY7715-EV (31%) groups, while heat-killed KCTC3108 and its EVs showed lesser reductions (52% and 97%, respectively) (Figure 2B). qPCR analysis showed that MYOG expression was most significantly increased by heat-killed HY7715 (138%), while MYH1 was moderately elevated (118%) (Figure 2C,D). HY7715-derived EVs had minimal effects on these genes. However, HY7715-derived EVs significantly suppressed the expression of the sarcopenia-associated genes Fbox32 and MuRF1 (to 55% and 68%, respectively) more strongly than heat-killed HY7715 (77% and 72%) or UA (88% for MuRF1) (Figure 2E,F). Heat-killed KCTC3108 and its EVs exerted modest effects (94% to 80% reductions). These findings suggest that both heat-killed HY7715 and its EVs promote muscle cell differentiation and mitigate muscle atrophy in vitro. Notably, heat-killed HY7715 had a stronger effect on myogenic differentiation, whereas the EVs were more effective at reducing atrophy-related markers.

### 3.3. Heat-Killed HY7715 and HY7715-Derived EVs Suppress Protein Degradation and Promote Myotube Formation in TNFα-Treated C2C12 Cells

**Figure 3:**
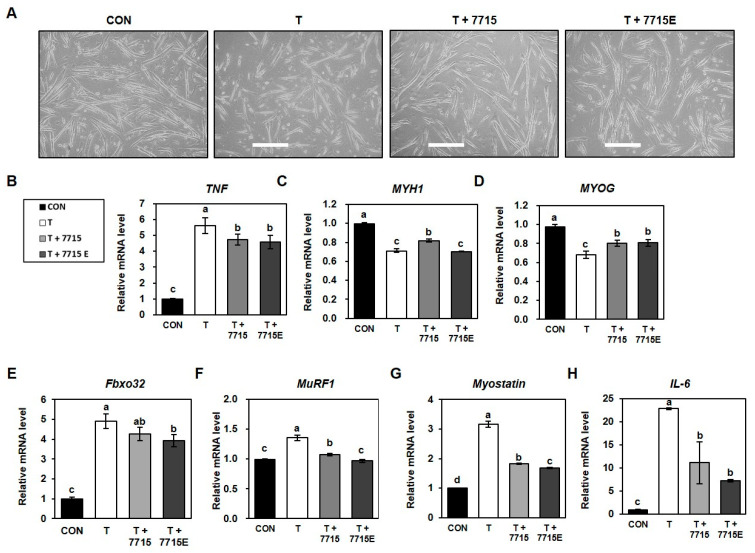
Effect of heat-killed HY7715 and its extracellular vesicles on myogenic differentiation and expression of sarcopenia-related genes in TNFα-induced C2C12 myotube atrophy. (**A**) Representative images of C2C12 myotubes after 5 days of differentiation in the presence or absence of TNFα (100 ng/mL), heat-killed HY7715 (10^6^ CFU/mL), or HY7715-derived EVs. Bar: 100 μm. (**B**–**H**) Relative mRNA expression levels of TNFα, MYH1, MYOG, Fbox32, MuRF1, myostatin, and IL6, measured by qPCR. Data are presented as mean ± SD (*n* = 4). Groups were compared using one-way ANOVA followed by Tukey’s multiple range test. Different letters indicate statistically significant differences (*p* < 0.05).

We examined whether heat-killed HY7715 or its EVs alleviate TNFα-induced muscle atrophy in C2C12 cells. Morphological analysis showed that TNFα (100 ng/mL) severely inhibited myotube formation, whereas treatment with heat-killed HY7715 or HY7715-derived EVs restored myotube fusion (Figure 3A). qPCR analysis revealed that TNFα increased TNFα expression by 562% compared to the control, and HY7715 and EV decreased the TNFα levels the TNFα-treated group by 49% and 32%, respectively (Figure 3B). Expression of MYH1 and MYOG was decreased by TNFα to 71% and 68%, respectively, compared to that in CON group. However, they were significantly increased by heat-killed HY7715 to 115% and 117% in TNFα-treated group; EVs also increased the expression of MYOG (118% of TNFα group), although MYH1 expression remained unchanged (Figure 2C,D). Conversely, TNFα treatment elevated the expression of atrophy-related genes Fbox32 (490%), MuRF1 (135%), and myostatin (317%) compared to that in the CON group. Heat-killed HY7715 reduced these to 87%, 79%, and 57% of the TNFα group levels, respectively. On the other hand, EVs further decreased them to 80%, 71%, and 53% of the TNFα group levels, respectively (Figure 3E–G). Additionally, IL6 expression increased to 228% with TNFα, but was suppressed to 49% of the TNFα group levels by heat-killed HY7715 and 32% of the TNFα group levels by EVs (Figure 3H). These results indicate that both heat-killed HY7715 and its EVs counteract TNFα-induced sarcopenic signaling by modulating the expression of myogenic and atrophic factors in C2C12 myotubes.

### 3.4. Heat-Killed HY7715 and HY7715-Derived EVs Increase Mitochondrial Biogenesis in TNFα-Treated C2C12 Myotubes

**Figure 4:**
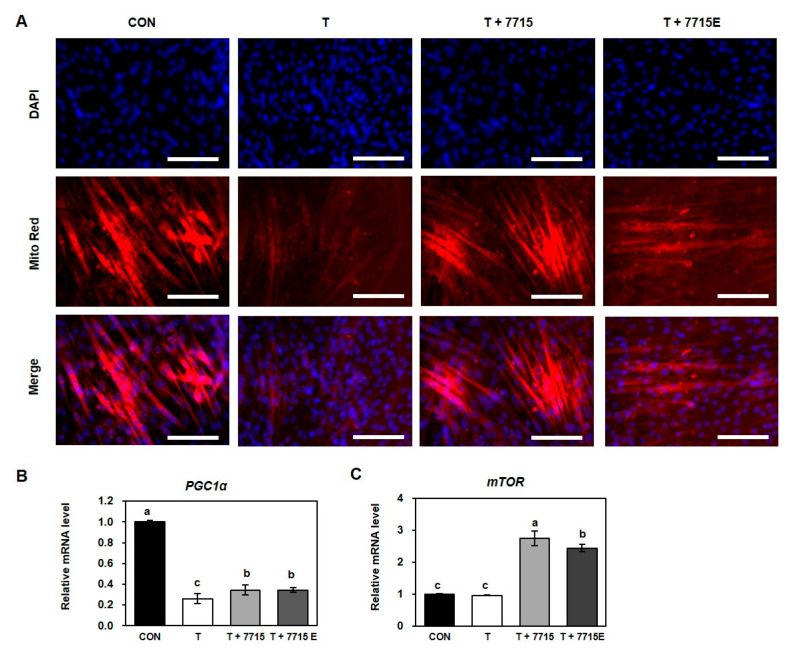
Effect of heat-killed HY7715 and HY7715-derived extracellular vesicles on mitochondrial activity and biogenesis-related gene expression in TNFα-induced C2C12 myotube atrophy. (**A**) Representative fluorescence microscopy images of C2C12 myotubes stained with MitoTracker Deep Red (mitochondria, red) and DAPI (nuclei, blue). Scale bar = 50 µm. (**B**,**C**) Relative mRNA expression of PGC1α (**B**) and mTOR (**C**) measured by qPCR. Data are presented as mean ± SD (*n* = 4). Groups were compared using one-way ANOVA followed by Tukey’s multiple range test. Different letters indicate statistically significant differences (*p* < 0.05). Scale bar = 100 μm.

Recent studies have reported that skeletal muscle atrophy is closely associated with dysregulation of mitochondrial biogenesis and metabolism [30]. To assess the effect of heat-killed HY7715 and its EVs on mitochondrial activity in TNFα-treated C2C12 myotubes, we conducted MitoTracker staining (Figure 4A). TNFα treatment markedly reduced mitochondrial activity, as shown by the decreased red fluorescence intensity compared with that of the control. Treatment with heat-killed HY7715 significantly restored mitochondrial activity, while HY7715-derived EVs had similar but slightly lower effects. We next analyzed the expression of genes related to mitochondrial biogenesis, including PGC1α and mTOR (Figure 4B,C). TNFα treatment decreased PGC1α mRNA expression to 26% of the control level, whereas treatment with heat-killed HY7715 and HY7715-derived EVs increased expression to 131% compared to that of TNF group. TNFα did not significantly affect mTOR expression compared to that of the control. However, heat-killed HY7715 and HY7715-derived EVs elevated mTOR expression to 286% and 253% of the TNFα group levels, respectively. These findings suggest that heat-killed HY7715 and its EVs enhance mitochondrial biogenesis and function, thereby potentially mitigating TNFα-induced muscle atrophy.

### 3.5. Heat-Killed HY7715 and HY7715-Derived EVs Improve the Tight Junctions Between Intestinal Cells

**Figure 5:**
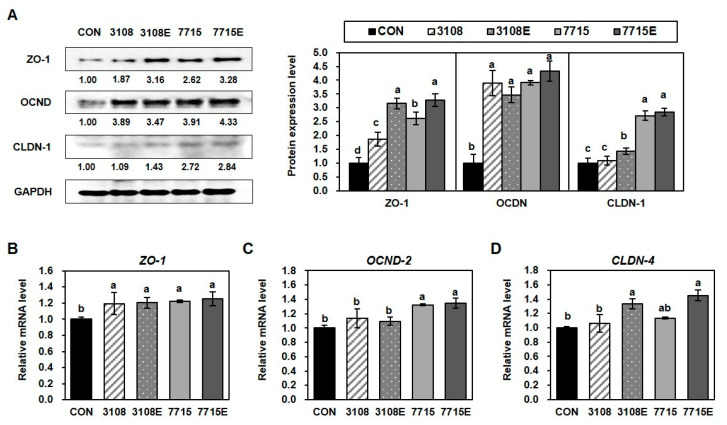
Effects of heat-killed HY7715 and HY7715-derived EVs on the expression of tight junction in Caco-2 cells. Caco-2 cells were differentiated under standard conditions (CON) or in the presence of 10^6^ CFU/mL heat-killed HY7715 (7715), heat-killed KCTC3108 (3108), or their derived extracellular vesicles (7715E, 3108E). (**A**) Protein expression levels of ZO-1, OCDN, and CLDN-1 assessed via Western blot. Expression was normalized to GAPDH and fold change relative to control is indicated. (**B**–**D**) Relative mRNA expression of ZO-1, OCDN-2, and CLDN-4, measured by quantitative PCR. Values are presented as mean ± SD (*n* = 4). Groups were compared using one-way ANOVA followed by Tukey’s multiple range test. Different letters indicate statistically significant differences (*p* < 0.05).

To evaluate the impact of heat-killed HY7715 and its EVs on intestinal barrier function, we assessed the expression of tight junction proteins in Caco-2 cells following 18 days of differentiation in the presence of each treatment (Figure 5A). Western blot analysis revealed that ZO-1 protein expression was significantly upregulated by heat-killed HY7715 (262%) and HY7715-derived EVs (328%) compared to that of the control. And treatment with heat-killed KCTC3108 and its derived EVs also increased ZO-1 expression, but to a lesser extent (187% and 316%, respectively). Notably, EV treatments exhibited stronger effects than their corresponding heat-killed probiotics. Similarly, occludin (OCDN) expression was markedly increased in the heat-killed HY7715 and HY7715-EV groups, reaching 319% and 733%, respectively, relative to the control. The expression of claudin-1 (CLDN-1) was also enhanced by both treatments, showing 272% and 284% increases with heat-killed HY7715 and HY7715-derived EVs, respectively. In parallel, gene expression analyses supported the protein-level findings (Figure 5B–D). mRNA expression of ZO1 was significantly elevated by all treatments to 119% (7715), 120% (3108), 122% (7715E), and 125% (3108E). OCDN2 gene expression was notably upregulated by heat-killed HY7715 and HY7715-derived EVs (132% and 135%, respectively). Interestingly, CLDN4 mRNA expression was significantly increased only by EV treatments, with HY7715-derived EVs inducing a 145% increase, compared to 133% for KCTC3108-derived EVs. These findings suggest that heat-killed HY7715strengthens intestinal epithelial barrier function by enhancing tight junction gene and protein expression, particularly via its EVs.

### 3.6. Heat-Killed HY7715 and HY7715-Derived EVs Ameliorate LPS-Induced Disruption of Tight Junctions in Intestinal Epithelial Cells

**Figure 6:**
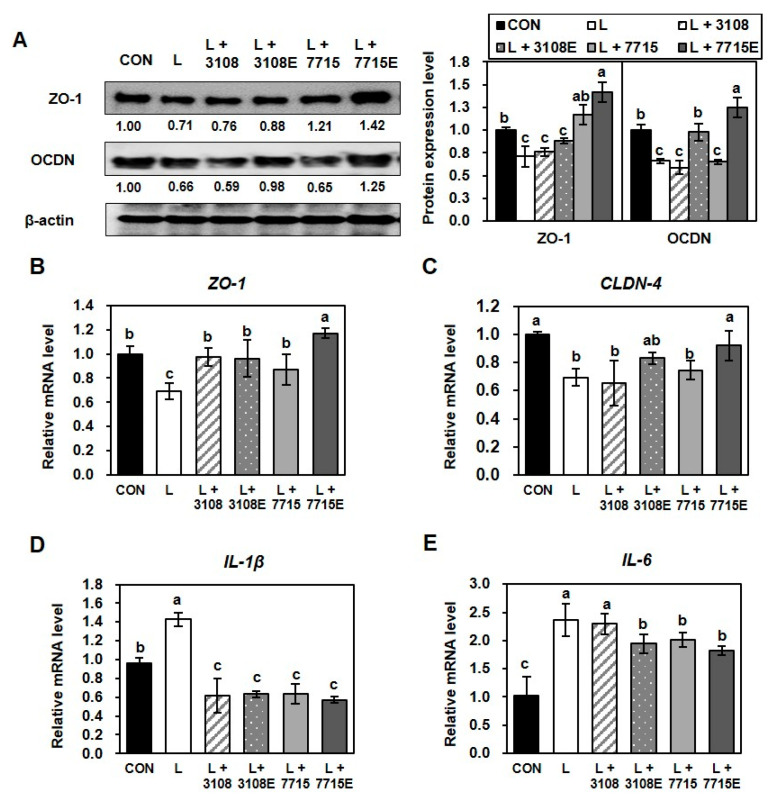
Effects of heat-killed HY7715 and HY7715-derived EVs on tight junction proteins and inflammatory gene expression in LPS-treated Caco-2 cells. Caco-2 cells were differentiated under normal conditions (CON) or treated with 1 ng/mL lipopolysaccharide (LPS, L) to induce inflammation. Cells were co-treated with 10^6^ CFU/mL heat-killed HY7715 (7715), KCTC3108 (3108), or their derived EVs (7715E, 3108E). (**A**) Protein expression levels of ZO-1 and occludin (OCDN) assessed by Western blot and normalized to GAPDH. Fold changes relative to the untreated control are shown. (**B**–**D**) Relative mRNA expression levels of (**B**) ZO-1, (**C**) CLDN-4, (**D**) IL-1β, and (**E**) IL-6 measured by qPCR. Data are presented as mean ± SD (*n* = 4). Groups were compared using one-way ANOVA followed by Tukey’s multiple range test. Different letters indicate statistically significant differences (*p* < 0.05).

Lipopolysaccharide (LPS), a key component of Gram-negative bacterial cell walls, is known to induce intestinal barrier dysfunction and inflammation by disrupting tight junctions and stimulating pro-inflammatory cytokine production [31,32]. To examine the protective effects of heat-killed HY7715 and its EVs against LPS-induced barrier damage, Caco-2 cells were treated with 1 ng/mL LPS, and tight junction protein expression was analyzed (Figure 6A). LPS exposure significantly reduced the protein expression of ZO-1 and occludin (OCDN) to 71% and 66% of control levels, respectively. Co-treatment with HY7715-derived EVs significantly increased ZO-1 and OCDN expression to 200% and 189% of the LPS group levels, respectively, indicating a strong protective effect. Heat-killed HY7715 also upregulated ZO-1 expression (164% of LPS group), but had a less pronounced effect on OCDN. In contrast, heat-killed KCTC3108 showed no restorative effect on either tight junction proteins, whereas KCTC3108-derived EVs modestly decreased OCDN expression to 89% of the LPS group levels, but had negligible effect on ZO-1. At the gene expression level, ZO1 mRNA expression was downregulated by LPS treatment to 69% of the control levels. This decrease was modestly attenuated by heat-killed KCTC3108 (141% of LPS group), KCTC3108-derived EVs (139% of LPS group), and heat-killed HY7715 (14% of LPS group), while HY7715-derived EVs fully restored ZO1 expression to 169% of the LPS group levels. Similarly, KCTC3108-derived EVs and HY7715-derived EVs significantly increased the CLDN4 gene value, which was reduced to 69% by LPS, and to 119% and 132% of the LPS group levels, respectively. Moreover, LPS treatment significantly elevated the expression of pro-inflammatory cytokines IL1B and IL6, to 143% and 236% compared to the control, respectively (Figure 6D). All postbiotic treatments effectively reduced these levels, with heat-killed HY7715 lowering IL1B and IL6 expression to 44% and 85% of those of the LPS group, respectively, and HY7715-derived EVs exerting the most substantial anti-inflammatory effect, reducing IL1B expression to 40% and 77% of the LPS group levels. Collectively, these results suggest that heat-killed HY7715 protects intestinal epithelial cells from LPS-induced tight junction disruption and inflammation, particularly via its EVs, by restoring tight junction-related factor levels and suppressing inflammatory cytokine production.

## 4. Discussion

Sarcopenia, characterized by the age-associated decline in skeletal muscle mass and function, is a major contributor to physical frailty and reduced quality of life in older adults [33]. This process involves both quantitative and qualitative alterations in muscle tissue, leading to impaired mobility and metabolic dysregulation [34]. Skeletal muscle mass begins to decline from approximately the fourth decade of life, with up to 50% loss observed by age 80. Given that muscle comprises nearly 60% of the total body mass, this loss profoundly affects whole-body metabolism and is linked to an increased risk of morbidity and mortality, particularly in individuals with metabolic disorders [5,35,36].

Emerging evidence suggests that the gut microbiota plays a key role in modulating skeletal muscle health, and interventions such as probiotics, prebiotics, and postbiotics may hold therapeutic potential [37]. Among these, postbiotics—non-viable microbial cells or their components with beneficial effects—have garnered increasing attention for their safety and stability. However, their efficacy in the context of sarcopenia remains largely unexplored. In this study, we investigated the postbiotic potential of heat-killed *Lactiplantibacillus plantarum* HY7715 and its EVs, a strain previously shown to improve muscle function and gut microbiota composition in aged mice [21].

Microbial EVs are known to carry biologically active molecules such as nucleic acids, proteins, and lipids that can enter host circulation and exert systemic effects [38]. EVs secreted by lactic acid bacteria (LAB), including HY7715, range in size from 20 to 800 nm and have been implicated in modulating gut and immune function [39]. In our study, EVs derived from HY7715 were characterized using nanoparticle tracking analysis, which revealed a particle concentration of 4.11 × 10^10^ particles/mL and modal and mean diameters of 127.6 ± 13.4 nm and 162.9 ± 7.5 nm, respectively. Given their ability to cross epithelial barriers and mediate inter-organ communication, we hypothesized that HY7715-derived EVs could influence skeletal muscle regeneration and differentiation [40].

To explore this, we first assessed the myogenic potential of heat-killed HY7715 and its EVs using C2C12 myoblasts. Our data demonstrated that both treatments significantly enhanced myogenic differentiation, with heat-killed HY7715 showing the highest induction of Myf5 expression and MYOG mRNA levels, surpassing that of the type strain KCTC3108 and ursolic acid, a known myogenic stimulator. Furthermore, in a TNF-α-induced atrophy model, both treatments of heat-killed HY7715 and HY7715-derived EVs restored the mRNA level MYOG to near-control levels, indicating their protective effect against inflammatory muscle damage.

Mitochondrial function is a key determinant of muscle health, particularly during aging. Using MitoTracker staining, we observed that heat-killed HY7715 notably enhanced mitochondrial activity, with stronger fluorescence intensity than EV-treated cells. Both treatments alleviated TNFα-induced mitochondrial dysfunction. Moreover, heat-killed -HY7715 significantly upregulated PGC1α and mTOR gene expression, critical regulators of mitochondrial biogenesis and cellular energy homeostasis. These findings suggest that heat-killed -HY7715 and its EVs may enhance muscle regeneration by promoting both myogenic differentiation and mitochondrial function.

Sarcopenia is also driven by enhanced protein degradation mediated through the ubiquitin–proteasome system [41]. FOXO-regulated genes such as Atrogin-1 (Fbox32) and MuRF1 (Trim63), along with myostatin, are known mediators of muscle atrophy [42]. Our results showed that HY7715-derived EVs effectively downregulated the mRNA levels of Fbox32 and MuRF1, as well as myostatin protein levels. Heat-killed HY7715 also attenuated their expression, but the effect was less pronounced than that observed with HY7715-derived EVs. Notably, both treatments exhibited anti-sarcopenic activity by attenuating the TNFα-induced upregulation of muscle atrophy-related genes. Additionally, HY7715-derived EVs more effectively reduced IL-6 expression than heat-killed HY7715 in the inflammatory model, suggesting stronger immunomodulatory capacity.

Given the proposed role of gut–muscle axis signaling in muscle maintenance, we also investigated the effects of heat-killed HY7715 and its EVs on intestinal barrier integrity. Tight junction proteins such as ZO-1, occludin (OCDN), and claudins (CLDN) are critical for maintaining intestinal permeability [43]. Both treatments significantly increased ZO-1, OCDN, and CLDN1 protein expression, along with the mRNA levels of OCDN2 and CLDN4. These effects were more pronounced with EV treatment, suggesting their superior efficacy in reinforcing the epithelial barrier. Moreover, under LPS-induced inflammatory stress in Caco-2 intestinal cells, HY7715-derived EVs highly restored tight junction protein expression and reduced that of pro-inflammatory cytokines IL-6 and IL-1β more effectively than heat-killed HY7715. LPS is known as a component of the outer wall of Gram-negative bacteria, mediates some inflammatory responses, and induces intestinal tight junction dysfunction [32]. These findings support the hypothesis that HY7715-derived EVs can maintain intestinal homeostasis under pathological conditions, which may in turn positively impact muscle health by mitigating systemic inflammation and improving nutrient absorption. Moreover, HY7715 and its EVs likely exert muscle-beneficial effects by modulating the gut environment (strengthening the barrier, reducing inflammation) and possibly by small-scale translocation or signaling that does not require bulk crossing of the barrier. Although these in vitro intestinal models (Caco-2 monolayers) suggest a protective effect of EVs on barrier function, further in vivo studies are needed to determine whether and how HY7715-derived EVs cross the barrier to reach distant tissues such as skeletal muscle. Recent studies have reported that bacterial vesicles can enter the circulation and affect the health of distant organs [40,44,45]. Based on this, since HY7715-derived EVs improve the integrity of the intestinal barrier, they may reduce the leakage of inflammatory molecules such as LPS into the circulation, thereby indirectly contributing to muscle health by reducing systemic inflammation via the gut–muscle axis. Furthermore, since there are currently no known unique strain-specific markers for HY7715-derived EVs, there is a need to further discuss the potential for future studies to label or identify these EVs in vivo. Indeed, recent studies are actively seeking specific molecular markers for bacterial EVs [26,46]. Furthermore, although we have demonstrated the bioactivity of HY7715-derived EVs, its precise functional components have not yet been elucidated. Specifically, it is not yet known which components (proteins, peptides, RNAs, metabolites, etc.) within EVs are responsible for the observed effects. Therefore, further proteomic or metabolomic analyses may be conducted to characterize the bioactive molecules of HY7715-derived EVs.

In summary, our findings demonstrate that both heat-killed HY7715 and HY7715-derived EVs enhance myogenic differentiation, support mitochondrial biogenesis, suppress muscle atrophy markers, and reinforce intestinal barrier function. These results suggest the potential of postbiotics as therapeutic agents to attenuate sarcopenia through regulation of the gut–muscle axis. While these in vitro results are promising, further studies are warranted to elucidate the precise molecular mechanisms underlying these effects and to validate them in vivo. Future research using animal models of age-related sarcopenia will help determine the translational potential of HY7715-derived postbiotics. In addition, since postbiotics are non-viable and do not proliferate in the intestine, they may be safer than probiotics for people with weakened immune systems or serious diseases. Therefore, evaluating the efficacy of these HY7715 postbiotics increases their potential value as probiotic products, and they can be utilized as additives to improve the physicochemical and sensory qualities of the product. Together, our results highlight HY7715 postbiotics—particularly EVs—as promising candidates for next-generation interventions targeting muscle aging. Their dual action on both skeletal muscle and intestinal epithelial health supports their potential use as a safe and effective strategy to delay or mitigate sarcopenia and promote healthy aging.

## 5. Conclusions

Our study demonstrates that heat-killed *Lactiplantibacillus plantarum* HY7715 and its EVs significantly enhance myogenic differentiation and mitochondrial biogenesis, and inhibit the expression of muscle atrophy markers such as Fbox32, MuRF1, and myostatin. These postbiotics also improve intestinal barrier integrity by upregulating tight junction protein expression and reducing the levels of pro-inflammatory cytokines under LPS-stress conditions. The dual action of HY7715 postbiotics on both skeletal muscle and the intestinal epithelium suggests that they act through the gut–muscle axis to counteract sarcopenia. These findings highlight the therapeutic potential of HY7715-derived EVs as next-generation postbiotics to support muscle health and healthy aging.

## Data Availability

All the data are contained within the article.

## Abbreviations

The following abbreviations are used in this manuscript:

HY7715	*Lactiplantibacillus plantarum* HY7715
EVs	Extracellular vesicles
LAB	Lactic Acid Bacteria
KCTC3108	*Lactiplantibacillus plantarum* KCTC3108
UA	Ursolic acid
PGC-1α	Peroxisome proliferator-activated receptor coactivator 1 alpha
mTOR	Mechanistic target of rapamycin
MYF5	Myogenic factor 5
MYH1	Myosin heavy chain 1
MYOG	Myogenin
Fbox32	F-Box Protein 32, atrogin-1
MuRF1	Muscle ring-finger protein-1, TRIM63
GAPDH	Glyceraldehyde 3-phosphate dehydrogenase
TNFα	Tumor necrosis factor alpha
LPS	Lipopolysaccharide
ZO-1	Human zonula occludens-1
OCDN	Occludin
CLDN-1	Claudin-1
IL-1β	Interleukin 1 beta
IL-6	Interleukin 6
